# Improving Explosive Detection Dog Performance in Sealed Parcel Searches Through a Targeted Training Intervention

**DOI:** 10.3390/ani16213513

**Published:** 2026-11-06

**Authors:** Paul C. Bunker, Emma Calabrese, Kathleen R. Sucher, Paola A. Prada-Tiedemann, Lauryn E. DeGreeff

**Affiliations:** 1 Chiron K9, Somerset, TX 78069, USA; 2 Department of Chemistry and Biochemistry, Global Forensics and Justice Center, Florida International University, Miami, FL 33199, USA; ecala017@fiu.edu (E.C.); ldegreef@fiu.edu (L.E.D.); 3 Forensic Analytical Chemistry and Odor Profiling Laboratory, Department of Environmental Toxicology, Texas Tech University, Lubbock, TX 79409, USA; ksucher@ttu.edu (K.R.S.); paola.tiedemann@ttu.edu (P.A.P.-T.)

**Keywords:** working dogs, dog olfaction, odor detection, operational performance, detection reliability, search behavior, handler–dog teams, training intervention, explosive detection, parcel searches

## Abstract

Explosive detection dogs are routinely deployed for parcel screening, yet realistic sealed-parcel searches may present challenges not captured by conventional odor-recognition training. In 2025, a black box study of ANSI/ASB Standard 092 identified substantially reduced detection of explosives in tape-sealed parcels, suggesting a potential training gap in this search scenario. This follow-on study evaluated whether brief, progressive training could improve dog performance in sealed-parcel searches. Thirteen operational dog teams participated in trials conducted in Lubbock, Texas, and Miami, Florida. Teams completed a baseline assessment using tape-sealed parcels containing 50 g of C4 or TNT, or distractors, followed by an odor recognition test (ORT) using controlled explosive odor presentations. Following the correction of identified odor-recognition deficiencies, teams completed a training intervention manipulating odor availability through variable parcel venting and requiring progression from odor-source recognition to trained final response. Performance was reassessed using the same parcel configuration. All teams detected C4 during the ORT, whereas only two teams detected TNT. Following intervention, overall sealed-parcel detection sensitivity increased by 35 percentage points (*p* = 0.0078). C4 detection increased from 38% to 69%, while TNT detection increased from 15% to 54%, although neither individual change reached statistical significance. The results demonstrate that odor recognition does not necessarily translate to effective source localization in sealed parcels and support progressive, operationally representative parcel-search training.

## Simple Summary

Explosive detection dogs are routinely used to search buildings, vehicles, and packages for explosives. However, recognizing and finding an explosive odor when it is trapped inside a sealed parcel can require different skills. This study examined whether focused training could improve dogs’ abilities to detect explosives in sealed parcels. Dogs first completed a baseline parcel search that demonstrated poor detection of target odors of C4 and TNT in taped boxes. The dogs then completed a controlled odor recognition test to determine whether they could recognize their trained explosive odors at higher vapor levels. All teams successfully detected C4, while only two teams detected TNT. The strong C4 performance in the controlled test, compared with poorer sealed-parcel performance, suggested that the challenge was not simply recognizing the explosive odor. Teams received additional TNT exposure, followed by a session of focused training on searching for explosives in parcels. After training, overall detection sensitivity increased by 35%, from 27% to 61%, representing a statistically significant improvement (*p* = 0.0078). C4 detection increased from 38% to 69%, while TNT detection increased from 15% to 54%, although neither improvement was statistically significant. These findings show that recognizing an odor does not necessarily mean a dog can successfully find it during a search. For operational parcel searches, training must go beyond odor recognition and prepare dog teams to locate explosives under realistic conditions where packaging, contents, and limited odor release can challenge detection.

## 1. Introduction

### 1.1. Background

The Explosive Detection Dog (EDD) is a common classification of working dogs employed by the military, law enforcement, customs, and, increasingly, the private sector for event and facility security. In the United States, EDDs are used as a primary screening tool for cargo in the airline industry to search parcels and packages in a variety of situations, and they have had success in locating explosive devices in cargo planes flying into the country [1]. EDD teams are also utilized to search indoor/enclosed environments such as buildings, residences, event venues, offices, and factories, as well as vehicles, including haulage trucks, trains, and aircraft. In all these search scenarios, an EDD team may encounter sealed parcels and packages that require searching.

Data regarding confirmed parcel-bound IEDs is restricted access, but it is known that thousands of incidents of suspicious packages are reported each year within the United States, with a number of recent incidents both in the United States and internationally highlighting that sealed parcels continue to be viable methods for delivering explosive or incendiary threats [2,3,4]. Sealed parcels pose unique challenges for detection by dogs, as the odor of the target changes in both quantity and quality depending on the parcel’s materials and contents [5]. These changes can challenge a dog’s olfactory capability if it has not been specifically trained for parcel detection.

Reduced quantities of available target odor, combined with the introduction of an odor matrix from the parcel’s construction materials and contents, can substantially alter the odor profile presented to the dog. Furthermore, adsorption or absorption of odorant molecules by packaging materials may reduce the availability of target compounds and alter their relative abundance [5,6]. Dogs must be able to generalize a target odor across the diverse presentation profiles in which the target may be encountered, including varying forms of concealment and packaging, rather than being conditioned solely to the odor of the pure, unconcealed target material [7]. In addition, odor leakage from a sealed parcel is generally confined to the seams and corners of the box, and if dogs have been trained to only search unsealed boxes, they typically search only the top opening.

Due to procurement and storage regulations regarding certain explosive training aids, including C-4 and TNT, dogs may not encounter these materials as often as those that are more readily accessible, such as smokeless powder. Gazit et al. (2005) demonstrated that dogs formed an olfactory search image for explosives [8], with TNT detection improving following frequent exposure and declining when TNT became less prevalent during training, highlighting the importance of continued reinforcement of individual target odors.

### 1.2. Problem Statement

A black box study, performed by Karpinsky et al. [9], evaluated the American National Standards Institute and American Standards Board’s (ANSI/ASB) Standard 92 that establishes requirements for the training and certification of explosive detection dogs [10]. The study utilized operational EDD teams that supported the hypothesis that standard training and assessment practices did not provide the dog with a clear understanding of how to search sealed parcels effectively. While the entire study examined a variety of search scenarios, the parcel search assessment posed the greatest challenge for the dog teams. In the study, ten cardboard parcels were prepared with typical commercial materials, such as packing and office supplies, with explosives added to two of the boxes and distractor (non-target) odors added to the other eight. The boxes were taped closed along the top and bottom middle seams.

The black box study results demonstrated that, among the 54 certified EDD teams participating across three locations in the United States, only 5% of the dogs detected the targets in tape-sealed parcels on Day 1 of the trial, rising to 67% on Day 2. However, when the same setup was repeated, with the tops of the boxes only folded over to keep them closed rather than being sealed by tape along the seams, with minimal or no box contents aside from the target or non-target odors, the dogs achieved 70% target detection on Day 1 and 100% on Day 2. Although performance on Day 1 was significantly lower for the tape-sealed boxes, the marked improvement on Day 2 suggests that reduced initial performance may have reflected limited experience with the sealed-parcel presentation rather than an inability to recognize the target odor. The findings of this study suggest that current training protocols do not adequately prepare detection dogs for the challenges associated with sealed-parcel searches, highlighting a potential operational gap between training and real-world search conditions.

Three behaviors were observed during the Karpinsky et al. study and were identified as not conducive to effective parcel searching. The dog teams were observed by experienced dog trainers, and the observations were consistent across all three study locations. The three behaviors are outlined below.

1. Dog nose punched the parcel. This behavior involved the dog punching the top of the parcel with its nose, typically at the top center. The behavior is thought to develop from training with parcels whose tops are folded and untaped. The dog learns that punching the top-middle of the parcel allows its nose to enter, thereby accessing the concentrated odor if the parcel contains its target. Dogs can be trained to detect specific levels of a target odor and to ignore larger or smaller presentations of the same target [11]. While this may be an advantage in some areas of dog detection, it is not when searching for explosives. Sealed parcels limit the availability of target odors because of limited openings for odor to escape and absorption by the parcel’s materials and contents. This suggests a failure to generalize detection at lower odor concentrations.

2. Dog scanned the top of the parcel only. This behavior likely develops when the folded parcel contains larger target amounts, so the odor permeates through the openings in the folds, and the dog does not need to engage in intense sniffing to detect the target. It can quickly scan along the top of the parcel and then move on. This results in parcels not being searched effectively and dogs missing any targets within a sealed parcel. In operational searches, parcels are often sealed rather than folded at the top, which makes the dog team’s search strategy inefficient. When a parcel is sealed, odor can escape through the seams and small openings at the corners. These areas offer the highest probability of detection. As a result of nose-punch behavior, dogs search only the top-middle of the parcel and do not detect odor at the corners or seams of a sealed parcel.

3. Dog immediately moved to the next parcel or searched objects within the room. If the dog has very little focus on searching the parcel beyond the two behaviors above, then because parcels are not deemed productive, it moves to more likely areas for detection, such as searching the area around the parcel array. This behavior reflects task abandonment and results from a conflict in reinforcement history; rooms are more likely to be productive than sealed parcels. Because the dogs receive more reinforcement during room searches in routine training, they will disengage from the parcel and begin searching objects in the room.

### 1.3. Study Aim

To further investigate the findings of the original black box Study, a follow-on training session was conducted at Texas Tech University (TTU) in Lubbock, Texas, and Florida International University (FIU) in Miami, Florida. The study was designed to determine whether a short period of focused training on sealed parcels could improve dog detection performance and, consequently, whether the poor performance observed during the original black box study was attributable primarily to limited training and familiarity with the sealed-parcel condition rather than an inherent limitation in dog detection capability. More broadly, the study sought to evaluate the extent to which targeted training could improve dog performance when searching sealed parcels and to provide insight into the adequacy of current training practices for this operational search scenario.

## 2. Materials and Methods

### 2.1. Study Design

All the protocols within this study were reviewed and approved by the Texas Tech Institutional Animal Care and Use Committee (IACUC) (Protocol 2023-1398), as well as the Florida International University IACUC (Protocol 201805). A repeated-measures design was used in this study involving Explosive Detection Dog teams, with their performance assessed before and after targeted training. A baseline assessment, in the form of a simple parcel search, was conducted to determine each dog team’s initial proficiency. The teams were then given remedial training for the deficiencies identified.

Following the baseline parcel assessment and before remedial training, an Odor Recognition Test (ORT) was conducted to determine the ability of the dogs to detect trained target odors in a controlled environment. Any deficiencies in the detection of target odors during the ORT were addressed first before the dog team progressed to the training intervention for parcel searching. The training intervention was specifically designed to improve the dog’s proficiency at detecting the target odor within a sealed box. After training was completed for each dog team, a final assessment was conducted with the same parcel search setup as the baseline to determine improvement in dog capability as a result of the training intervention.

All assessments were conducted under controlled indoor conditions, using standardized search environments, target odors, distractors, and evaluation procedures to ensure consistent, reliable data collection across all teams, as described below.

#### Dog Teams

Two separate training trials occurred at different locations with different teams. The first trial occurred in Lubbock, Texas, in January 2026 with seven teams. The second trial occurred in Miami, Florida, in March 2026 with six teams. For both locations, there was a mix of active law enforcement dog teams and teams from private contractors or training agencies. All teams were certified for explosive detection, and the handler experience was greater than four years for most teams that participated.

### 2.2. Materials

C4 and TNT, purchased from OMNI Explosives (Crawfordsville, AR, USA), were selected as the explosives for use throughout this study based on previous use in Karpinsky et al. in parcel searches [9]. Distractors, utilized in the ORT and parcel searches, were obtained locally for each trial (e.g., dryer sheet, Sharpie, nitrile glove). Both explosives and distractors were placed in cotton bags (4″ × 2-3/4″—10 cm × 5 cm, Pacific Midwest, Grainger) throughout the study.

Cardboard boxes used were sourced from unrelated deliveries to Texas Tech University and Florida International University, the locations of the trials. The boxes selected for use varied in sources, shapes, and sizes, though they were within the same size range to avoid too much variation. All the boxes were structurally sound and could have their tops closed and sealed with standard packing tape. For use in the parcel searches, various packing materials were collected from volunteers from each location and placed inside the boxes for the trial (magazines, clothing, paper, bubble wrap, etc.).

Twelve plastic ammunition boxes (Plano 1212 Field Ammo Boxes, Academy, Katy, TX, USA) were used for the ORT setup; each box had ten holes drilled into the top to allow odor to escape. Each ammunition box contained a cleaned house brick (Home Depot) to reduce movement of the boxes during the search.

### 2.3. Baseline and Post-Training Parcel Assessments

**Figure 1:**
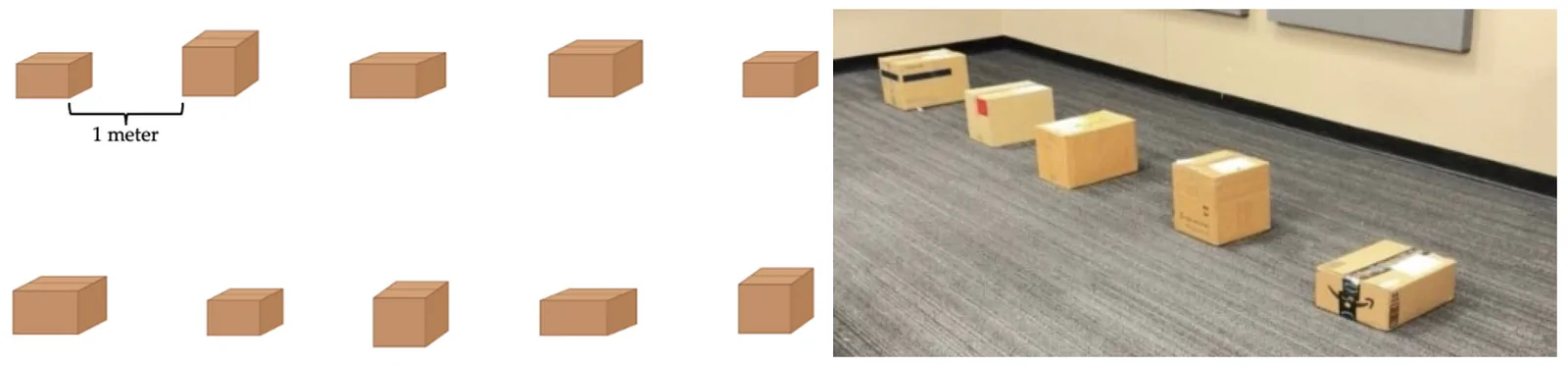
Diagram (**Left**) and photo (**Right**) of the parcel assessment line-ups, consisting of a total of 10 boxes placed 1 m apart, used for the baseline and post-training parcel assessments.

The baseline and post-training assessments followed the ANSI/ASB Standard 092, which includes protocols for conducting baggage/parcel assessments to determine a dog team’s capabilities in locating explosives hidden within sealed bags/parcels [10]. In accordance with Standard 092, the assessments used ten sealed boxes, placed in two lines of five with 1 m spacing between each, to allow the dog teams to work independently and systematically through each parcel (Figure 1). All boxes contained packing material; one box contained 50 g of C4, one box contained 50 g of TNT, and the remaining eight each had a distractor. The parcels were prepared 1 h before the baseline and post-training parcel assessments. The target locations within the parcel array were randomized for each search assessment to ensure that the assessment accurately reflected the dog’s detection capabilities and eliminated any potential influence of memory, visual cues, handler bias, or predictable target placement. However, a target odor was not placed adjacent to the other target odor to avoid any potential influence.

Assessments were completed using a single-blind evaluation design, in which the evaluator knew the target odor placements (locations and quantity) but the handler did not. While the handler received search parameters, they were not informed of the desired outcomes. With this design, the evaluator was able to observe the team’s performance, compare the handler’s reported findings with the actual target odor placements, and document the results objectively. The blinded test design is especially critical for parcel searches due to the potential for a handler’s subtle expectations and cueing to influence the dog’s behavior while working through a controlled environment containing similar items.

### 2.4. Odor Recognition Test (ORT)

**Figure 2:**
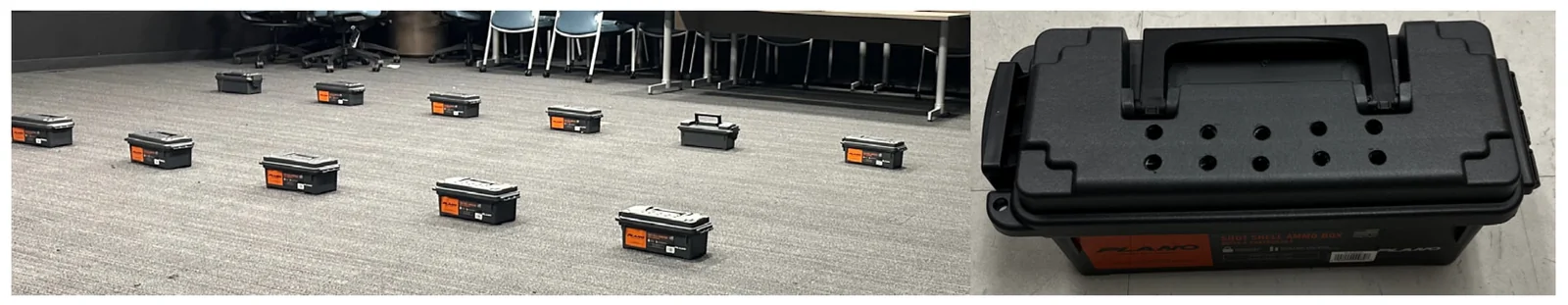
(**Left**) Line-up of twelve prepared ammunition boxes for the ORT, each separated by 1 m. (**Right**) Close-up of the ammunition box lid with ten holes drilled into it.

The ORT was conducted immediately after the baseline parcel search and before beginning the intervention training. The purpose of the ORT was to (1) determine if the dog had a true deficit in odor recognition, or (2) if the dog’s search performance in the baseline parcel search was impacted by either the sealed packaging, the search strategy, the accessibility of the source, or handler factors. The ORT consisted of a controlled line-up of target explosive odors, distractors, and blanks within twelve plastic ammunition boxes (Plano 1212 Field Ammo Boxes, Academy). Within the lineup, there was one ammunition box containing 50 g of C4, one with 50 g of TNT, and the rest had either a distractor, an empty cotton bag (match blank), or nothing (blank). Each ammunition box contained a house brick to avoid being moved by the searching dogs. The order of the ORT lineup was randomized for each team, and the tops of the boxes were wiped with a paper towel between teams to remove any deposited slobber. The ammunition boxes were placed in a lineup of two rows with each can being 1 m apart (Figure 2).

#### TNT Training

Based on the ORT results, which demonstrated a lack of TNT detection, the teams were provided an opportunity for additional TNT exposure and training before proceeding to the sealed-parcel intervention. Each dog team was given a training session using the same ammunition boxes used in the ORT. Line-ups of three boxes were used, with one box containing 50 g of TNT in a cloth bag and a brick, and two boxes containing just a brick. The dogs were provided three opportunities to sniff the ammo boxes, and when they sniffed the one containing TNT, the behavior was marked and rewarded. Between each trial, the boxes changed position within the lineup. The dog was then provided an opportunity to search the line of boxes, and if they responded to the ammo box containing TNT with a trained alert, the training was completed. If the dog did not alert to the TNT odor, the three trials with mark on sniff were repeated, and then the dog was again allowed to search, and if it demonstrated a TFR at the TNT, the training was completed.

### 2.5. Training Intervention

#### 2.5.1. Training Methodology

Based on the three observed behaviors from the Black Box study outlined above, a one-day training plan was developed to address and improve detection capability. While this was only a one-day event, it was considered sufficient to demonstrate that focused training could improve the dog’s capabilities, even in a short period.

The training plan was built on the concept of progressive exposure to variable presentations of the target odor and included increasing complexity by reducing the available odor and using sealed parcels. Using positive reinforcement for correct behaviors forms an association between parcels and the target odor. A marker, or a signal, usually audible, conditioned with reinforcement to serve as a bridge between a behavior and a reward [12], was used to communicate to the dog when the correct behavior was achieved. Before the training event started, it was confirmed with the handlers that they had a conditioned marker for use with the dog. The type of marker and reinforcer the handlers were expected to use was not specified by the evaluators, though most, if not all, teams utilized the phrase “yes” followed by the presentation of a toy, revealed as close as possible to the location of the target odor to ensure reinforcement. Throughout the study, handlers were permitted to reward and reinforce their dogs following the correct detection of a target within a session or training program.

Initial exposures to the target odor emanating from the parcel were marked when the dog sniffed at the location from which odor could escape. This is termed “mark on sniff” and provided the dog with clear communication that sniffing the corner of a parcel seam and detecting the target odor are desired behaviors. As the training progressed, the dog would be expected to give a trained final response (TFR). A TFR demonstrates when the dog adopts a predetermined (trained) body position/behavior that indicates to the handler it has located the target odor. Typically, this is a sit, down, or stand-and-stare, but can be other behaviors such as barking and scratching for other detection roles.

In this study, “threshold” refers to the quantity and composition of volatile, target-related compounds accessible to the dog outside the parcel during sealed-parcel searches. During training, the odor was physically manipulated by changing the number of openings through which the VOCs could escape, while maintaining 50 g of explosive material in the target parcels. The vapor concentrations were not measured; consequently, these physical changes represent differences in presumed odor availability and should not be interpreted as measured concentrations or dog detection thresholds.

#### 2.5.2. Training Workflow

The intervention consisted of six progressive trials manipulating odor availability through systematically decreasing vapor escape from the parcels. The training was conducted in a different space from the baseline parcel assessment and used cardboard boxes and materials that were similar but not associated with the baseline assessments. Each lineup consisted of three parcels: two blank parcels with only packing materials (clothing, magazines, etc.) and one with a target explosive. Six target boxes were prepared for the six training lineups, each containing 50 g of target explosives, three with TNT and three with C4. All of the parcels were prepared by adding packing materials and then sealed with tape 1 h before the first training event.

**Figure 3:**
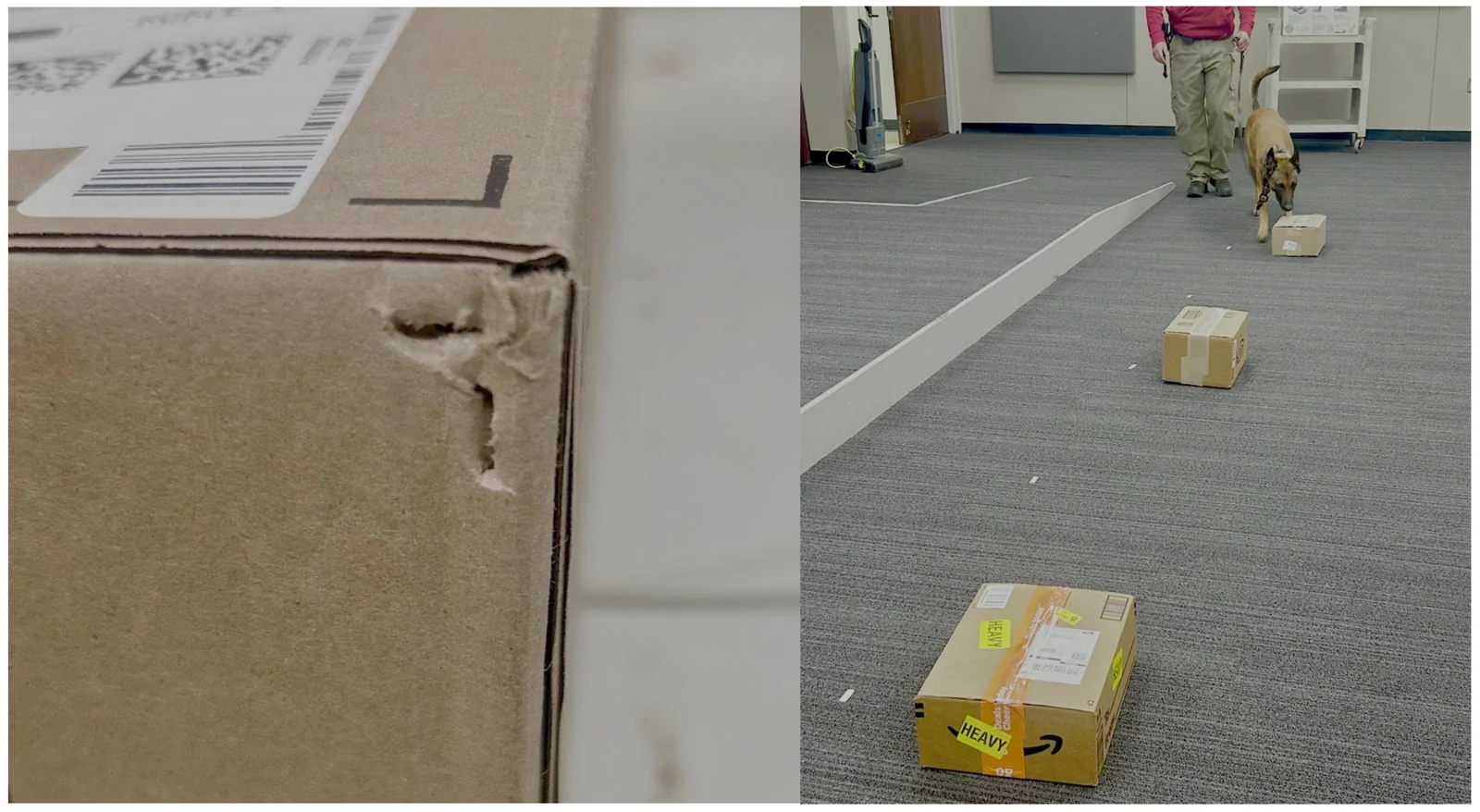
(**Left**) Example of a cardboard box with holes in the top corner as used in the training session (Parcel C). (**Right**) Detection team searching a lineup during the intervention training.

The quantity of available odor was controlled via holes of varying sizes made in the top corner of the parcels (Figure 3). Allowing the most available odor were the boxes with six holes, each 2 mm wide and 2 cm long (Parcel A), followed by boxes with four holes, each 2 mm wide and 2 cm long (Parcel B), and the least odor was released by the boxes with two holes, each 2 mm wide and 1 cm long (Parcel C).

A lineup search was used for training. The lineups consisted of randomized ordering of three parcels, one containing the target odor and two blank parcels, placed in a row with 2 m of space between them. The sequence for each dog team was (1) C4 in Parcel A, (2) C4 in Parcel B, (3) C4 in Parcel C, (4) TNT in Parcel A, (5) TNT in Parcel B, and (6) TNT in Parcel C.

For Trials 1, 2, 4, and 5, the handlers were informed of the holes in the parcels and instructed to mark when the dog was sniffing at the location of the holes and a Change in Behavior (CoB) was observed. A CoB is a term used to describe the body language that a dog demonstrates when it detects the target odor, but before it gives its TFR [13]. The CoB is the first indication that the dog has detected the target and marks the beginning of the detection chain, prompting it to follow any available plume and locate the target’s source. For Trials 3 and 6, the handlers were told to wait for a TFR by the dog, after which point the dog was deemed ready to move on to the post-training parcel search.

### 2.6. Data Collection

Evaluators collected data throughout the trials on the dog team’s performance. The major performance measures collected during the assessments were true positive, false positive, true negative, and false negative. Each of these metrics provides objective measures of how well the dog team detects the target within a parcel, and they are commonly used in operational testing and evaluation, as well as in research studies. The terminology is outlined in Table 1 below. In the instances of True and False Positives, the result was only recorded as such if the handler stated the target was present. If the dog gave a response or CoB and the handler did not state that the target was present, it was not recorded.

A True Positive, or positive alert, indicates a dog’s accuracy at locating a target in a lineup. A False Positive, or false alert, can determine how well a dog has discriminated among odors and whether the false alerts are influenced by handler cueing, residual odors, environmental contamination, or excessive expectation of success. A True Negative indicates a dog’s ability to ignore non-target odors. A False Negative, or miss, is important when evaluating the overall effectiveness of a dog in an operational environment, as it may indicate undetected safety threats. There are multiple ways to view the potential underlying causes of misses, including deficiencies in training, problems with identifying/modeling the odor, no odor available, environmental conditions that affect detection, and factors related to the handler’s accuracy or reliability.

By monitoring a team’s true positives, false positives, true negatives, and false negatives, various performance metrics (e.g., detection rate, precision rate, true detection rate, false response rate) were evaluated and used to compare dog performance before and after training, as well as to evaluate the effectiveness of the progressive sealed-parcel training program.

### 2.7. Data Analysis

Detection sensitivity was calculated as the proportion of target presentations correctly identified by the dog teams, expressed as sensitivity = TP/(TP + FN), where TP represents true positives, and FN represents false negatives. Similarly, specificity was calculated as the proportion of non-target presentations correctly rejected, expressed as Specificity = TN/(TN + FP), where TN represents true negatives, and FP represents false positives. Changes in sensitivity and specificity between baseline and post-training assessments were expressed as absolute percentage-point differences.

Dog detection performance was compared between baseline and post-training parcel assessments using paired statistical analyses to determine the significance of the change in the number of explosive targets correctly detected by each dog team, with two possible target detections (C4 and TNT) per assessment. Due to the small sample size and non-normality in the data, overall improvement was evaluated using a two-sided exact sign test comparing the number of teams demonstrating improved versus decreased detection performance. Because the test discards unchanged outcomes (i.e., ties), teams with unchanged performance were excluded from the calculation of the test statistic.

Changes in the detection of individual analytes, TNT and C4, were evaluated using two-sided exact McNemar’s tests for paired binary outcomes. Detection sensitivity was calculated as the proportion of target presentations correctly identified, and changes in sensitivity were expressed as absolute percentage-point differences between baseline and post-training assessments. False-positive responses were evaluated separately to characterize potential changes in detection specificity.

Statistical significance was established at α = 0.05. Exact *p*-values were reported for all statistical comparisons. A prospective power analysis was not conducted because the study was designed as a preliminary proof-of-concept investigation using available operational dog teams.

## 3. Results

### 3.1. ORT Results

**Figure 4:**
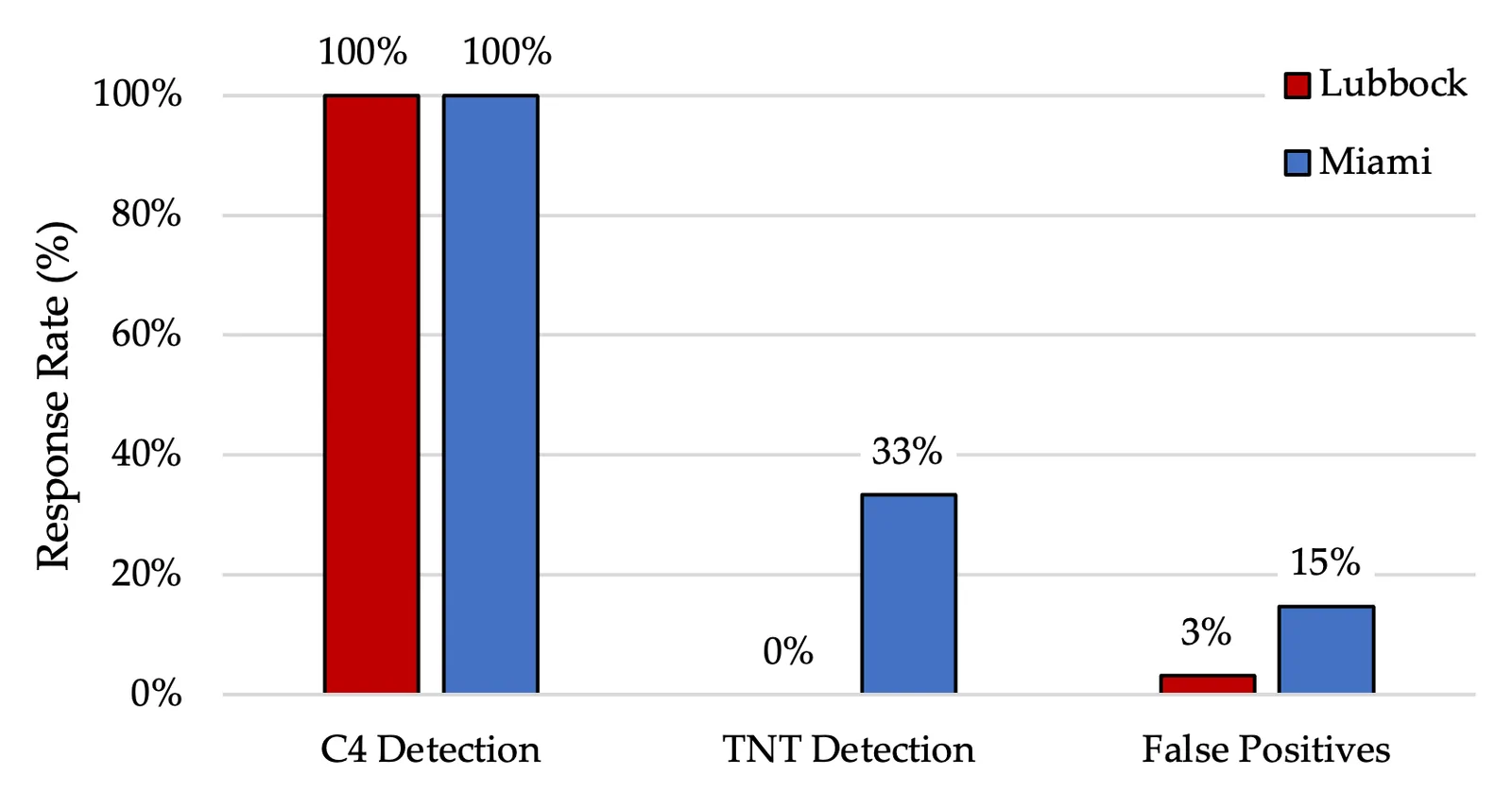
Rate of responses in an odor recognition test (ORT) for dogs that attended parcel training, separated by trial location (*n* = 7 for Lubbock; *n* = 6 for Miami).

The intention of the ORT was to confirm the dogs’ ability to detect trained target odors in a controlled environment and was carried out after the baseline test and before the training. Figure 4 demonstrates that there was a considerable deficit in the detection of TNT across both trial locations, with only two teams in Miami identifying TNT. Details of the alerts for each dog team can be found in Appendix A. The strong performance of all teams in detecting C4 during the ORT indicates that the poor C4 detection observed in the sealed-parcel searches was not attributable to the target odor itself or an inability to recognize C4. Rather, the discrepancy between performance in the ORT and the sealed-parcel searches suggests that factors associated with the parcel-search scenario, including the presentation and concealment of the target, may have influenced detection performance.

### 3.2. Parcel Assessments: Baseline and Post-Training

The data across the baseline and post-training parcel assessments for both trial locations are included in Table 2, with the details of the alerts for each dog team available in Appendix A. Overall, detection sensitivity increased for both explosive targets and at both locations following intervention training. However, false-positive responses also increased.

**Figure 5:**
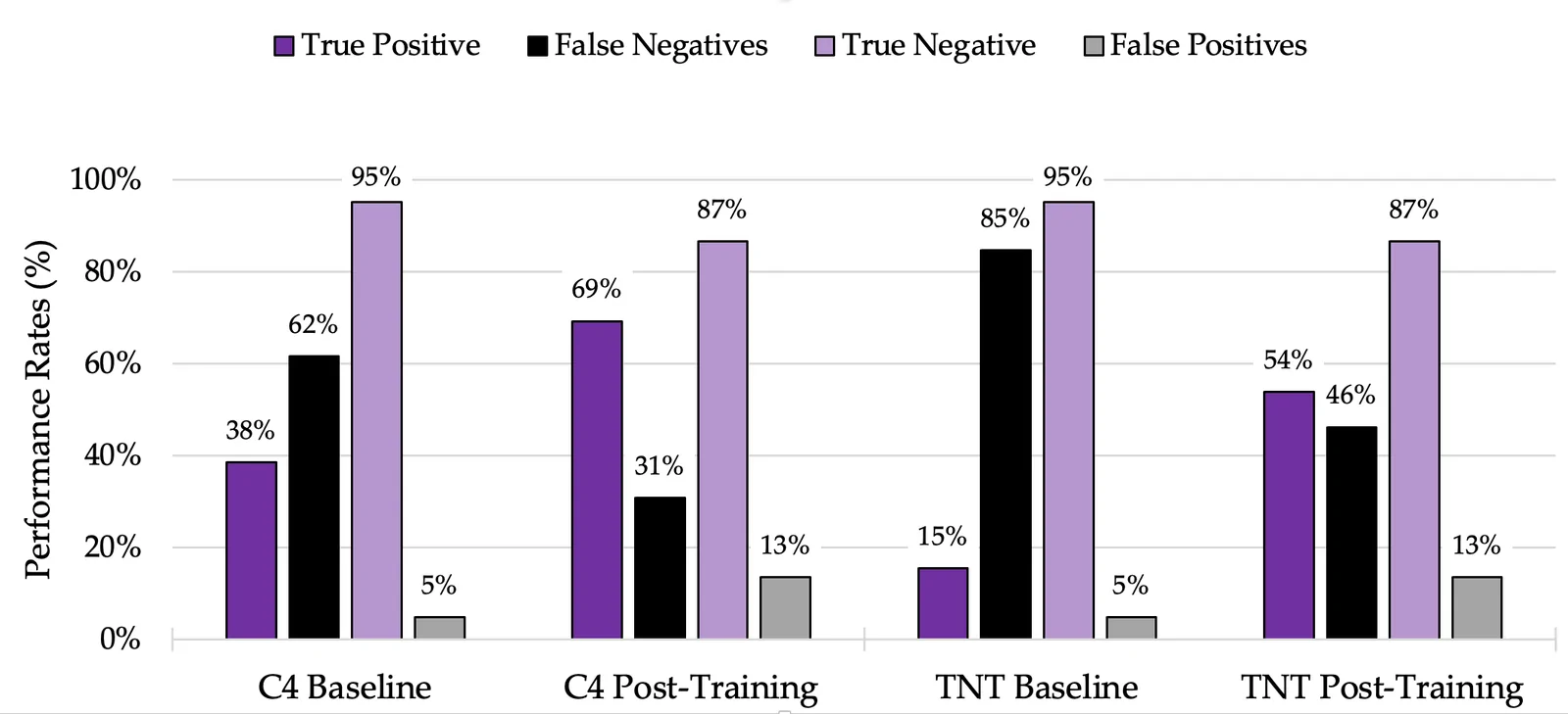
Combined performance of C4 and TNT detection for the Lubbock and Miami teams across the Baseline and Post-Training Parcel scenarios (*n* = 13).

Figure 5 gives the detection rates of explosives (TNT and C4) in sealed parcels for all dogs, comparing baseline and post-training. Overall detection sensitivity increased from 27% (7/26) at baseline to 61% (16/26) following training, representing an absolute improvement of 34.6 percentage points. Of the 13 dog teams evaluated, eight demonstrated improved detection performance, five remained unchanged, and none demonstrated decreased performance. An exact paired sign test indicated that the overall improvement was statistically significant (two-sided *p* = 0.0078).

Detection of C4 increased from 38% (5/13) at baseline to 69% (9/13) following training, representing an improvement of 31%. TNT detection increased from 15% (2/13) to 54% (7/13), an improvement of 38%. Exact McNemar’s tests indicated that neither the improvement in C4 detection (*p* = 0.125) nor that in TNT detection (*p* = 0.0625) was individually statistically significant at α = 0.05, Table 3. However, both explosive targets demonstrated substantial numerical improvements, and no dog team that correctly detected a given explosive during the baseline assessment failed to detect that explosive during the post-training assessment.

**Figure 6:**
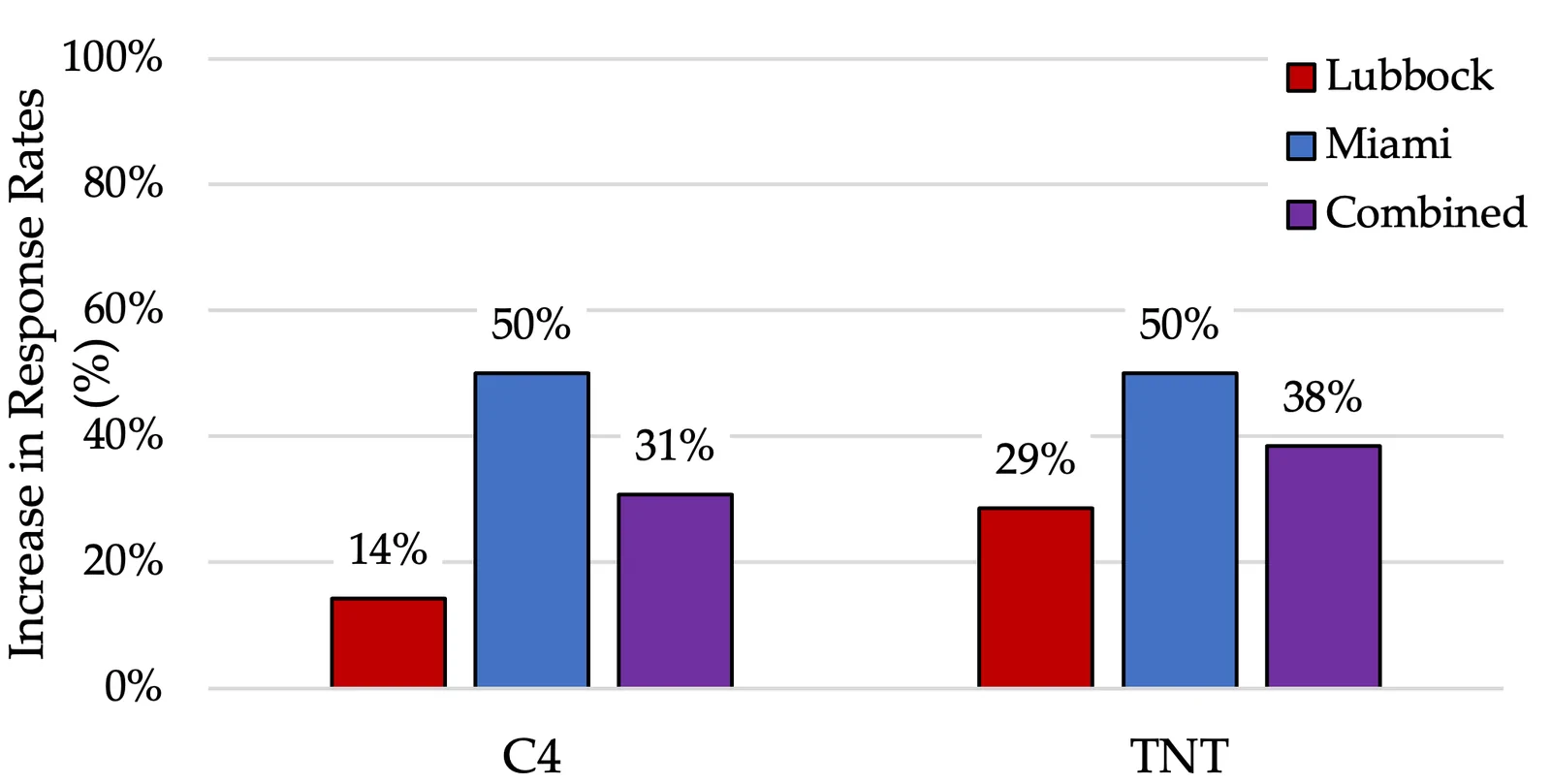
Percent increase in detection of C4 and TNT after the parcel training session (Lubbock *n* = 7; Miami *n* = 6; Combined *n* = 13).

As illustrated in Figure 6, improvements in detection sensitivity were observed at both trial locations, although the magnitude of improvement differed between explosives and locations. The Lubbock teams demonstrated increases of 14% and 29% for C4 and TNT, respectively, while the Miami teams demonstrated increases of 50% for both explosives. These results indicate that detection performance improved following the intervention across both locations, despite the limited training period.

Notably, each dog received less than 30 min of structured training, including additional imprinting with TNT, prior to the post-training evaluation. The poor TNT recognition observed during the ORT was not anticipated, and some of the available training time was allocated to ensure that dogs recognized TNT before proceeding with the sealed-parcel intervention. Consequently, the observed improvements reflect the combined effects of additional TNT exposure and focused parcel-search training.

#### 3.2.1. Training Progression Observations

Throughout the training phase, the dogs improved their search behavior. Scanning across the top of parcels was an issue at the start of the event; by the end of the training, this had stopped, and the dogs searched methodically. There was reduced nose punching of the parcels; instead, the dogs systematically searched all sides, including the seams. Disengagement from the parcels decreased. The sniffing intensity observed was greater in terms of both duration and persistence, with greater focus on the seams and corners than on the top of the parcel.

#### 3.2.2. Increased False-Positive Responses

The increase in false alerts may reflect a sensitivity–specificity tradeoff produced by the intervention. The training intervention reinforced increased sniffing behavior of sealed parcels and responses to lower thresholds of available target odor. This may have reduced the dogs’ response criteria, making them more likely to respond when odor information was weak or unclear. The limited training period with high odor expectation may also have increased target anticipation. Any continuance of training should include proofing the dog off ambiguous odor signatures and determining whether increased sensitivity can be retained while restoring specificity.

## 4. Discussion

### 4.1. Key Findings

The findings of this study indicate that a brief, structured training intervention was associated with measurable improvements in dog detection performance during sealed-parcel searches. Overall detection sensitivity increased from 27% at baseline to 61% following training. Eight of the thirteen teams demonstrated improved detection, five remained unchanged, and none demonstrated decreased performance. This overall improvement was statistically significant (*p* = 0.0078), supporting the potential value of targeted training for improving detection performance in operationally representative parcel searches.

Although improvements were observed for both C4 and TNT, neither explosive demonstrated an individually statistically significant change. C4 detection increased by 31%, while TNT detection increased by 38%. These improvements are operationally relevant, particularly given the limited duration of the intervention. However, the small number of dog teams limits the statistical power available to detect changes for individual explosive targets. The results therefore provide stronger statistical evidence for an overall improvement in detection performance than for improvements associated with either explosive independently.

The results of the ORT demonstrated that the dogs had moderate recognition of target odors, in particular the C4, which had been used in the baseline assessment. In analyzing the sensitivity, determined as the percent ratio of the true positives to the total number of targets, the dogs achieved 58% sensitivity in the ORT, which was considerably higher than that in the baseline parcel assessment (27% sensitivity). The difference suggests that the target odor recognition and detection may be influenced by the conditions in which the odors are presented. The ORT and the sealed-parcel assessments did differ in the presentation of the target odors, and the results are not able to establish if the reduction in parcel performance was attributable to odor availability, the search behavior, olfactory sensitivity, or a combination of these factors. Although the sensitivity during parcel search assessment did increase to 62%, and while this improvement is consistent with the potential benefit of targeted training, the uncontrolled study design does not provide the contribution to be distinguished from additional odor exposure, repeat testing, or increased familiarity with the assessment conditions.

The study demonstrated that training should be an ongoing process, emphasizing that odor detection skill is not a fully developed trait achieved during the primary imprinting phase. Consequently, the process of recognizing actual odors, especially in complex working conditions, requires constant practice and re-training. Therefore, training should comprise varied procedures and approaches to odor delivery, including different packing materials, various containment systems, and distractor odors.

The post-training observations demonstrated a shift from rapid, unfocused scanning of the parcels to systematic, deliberate search behavior. The dogs exhibited intense sniffing, duration, frequency, and persistence, resulting in a more engaged search pattern. These behaviors may provide opportunities for odor acquisition where the VOCs escape via gaps or imperfections in the packaging. Changes in the search behavior could have increased the likelihood of encountering the target odor. However, the study did not quantify the odor concentrations or olfactory detection thresholds. Therefore, these behavioral changes cannot establish detection performance independent of other factors such as repeated testing and exposure to the assessment conditions.

The reduction in nose-punching and scanning search behaviors showed that the dogs adapted their search behavior even within a short period. In addition, the dogs focused on the parcels rather than the environment and would search all four sides and the tops of the parcels.

These findings support the hypothesis that poor performance in sealed parcel searches results from training history and search strategy. The findings do not exclude the potential that limitations in odor availability or a dog’s olfactory sensitivity may also influence detection performance. Because the study used a pre–post design, the improvements observed cannot be attributed just to the training procedures. Nevertheless, the findings do suggest that the detection performance in parcel searches may be modifiable in a short period of time, supporting further controlled investigation.

### 4.2. TNT Recognition Issue

An important finding of this research was a lower success rate for dog teams in TNT detection. During the baseline and ORT assessments, TNT was detected less often than other odors, indicating that few of the dogs in the study recognized this target under the assessment conditions. Even though dogs improved their performance after the intervention training, preliminary findings indicate a capability gap in applying trained skills under different conditions.

These findings may indicate limitations in a dog’s previous training experience, the target odor introduction, or continued exposure. Informal interviews with the handlers revealed limited access to TNT as well as perceived difficulty in detecting the TNT odor [14]. These findings may indicate that low odor exposure experience during maintenance training is a limitation for the canines. Therefore, dogs may have difficulty identifying explosives they do not encounter often, especially TNT, reducing their detection capability and rate. However, because the frequency of TNT exposure prior to the study was not measured, the influence on performance cannot be reported.

Having genuine TNT may also affect the dogs’ proficiency in performing their tasks. There may be safety-related restrictions and existing regulations that hinder direct access to TNT, prompting organizations to rely on substitutes. The substitutes may not accurately capture the odor of the real TNT. The inability to generalize from one source of TNT to another may be a contributing factor in detection capability. Dogs that encounter only a limited number of training samples during routine training may not generalize to the same explosive type when it is altered by packaging, environmental contamination, production differences, or impurities [15].

While teams also had limited access to C4, plasticized explosives, such as C4, have an added, high-vapor-pressure taggant (dimethylnitrobutane, DMNB) that increases the ease of detection compared to the low-vapor-pressure TNT [16]. Training that excludes lower-threshold odor levels may leave dogs unable to detect low-vapor-pressure targets, such as TNT, at very low concentrations in challenging operational conditions. Because this study did not measure individual canines’ detection thresholds or previous training experience, the influence of these factors remains uncertain. The results may be related to a threshold problem rather than to an outright failure of odor recognition. It is possible that the configuration of a sealed parcel has reduced odor availability to a level that is now close to the threshold for dog detection. Further research would be needed to establish the limitations of odor recognition from low odor availability or detection sensitivity.

It appeared that the reason for the reduced TNT performance did not have to do with a single cause. Rather, it may result from the interaction of multiple factors, such as the lack of variance in training samples, the frequency of training maintenance sessions, odor availability, and a low detection threshold. All these factors must be considered to achieve desirable results.

### 4.3. Odor Availability in Sealed Parcels

A key challenge of sealed parcel searches is associated with the limited amount of available target odor. In typical training situations, target odor sources release sufficient vapor for detection by a dog. However, in sealed parcels, vapor is reduced by the accumulation of packages, absorbent substances, airtight seals, and the properties of the parcel itself. This results in dogs needing to locate the target under very difficult conditions, making parcel searches a demanding operational environment unless specifically trained for them.

Reduced odor thresholds are a challenge for those dogs not trained or experienced in detecting targets in these situations. Especially if the dog’s typical training uses odor sources with much higher vapor levels. Thus, there is a need to implement a system of progressive exposure to increasingly challenging presentations of the target odor to achieve a high level of detectability in low-odor-threshold situations.

Because there is potential reduction in target odor availability in sealed parcels, the importance of olfactory sensitivity and search strategy must be considered. The study did not measure the concentration of target VOCs, and it remains uncertain if the observed detection failures were directly associated with low odor availability, inadequate odor recognition, search behavior, or a combination of these factors. Detection depends on the dog’s ability to identify the target odor and to cover all areas where odors are potentially present.

Detection dogs need to search in a methodical, systematic manner rather than conducting a rapid, irregular search pattern; this will allow more opportunities for odor acquisition that lead to detection. The handler may influence the working process by maintaining an effective and efficient search pace, so that the dog is not unnecessarily influenced, and by giving the dog an opportunity to investigate all areas where only a small amount of odor is present. However, the effectiveness of these practices does require further investigation under controlled conditions.

### 4.4. Operational Implications

This study demonstrated important implications for dog teams that may be required to screen parcels in operational settings. In real-world environments, detection dogs may be required to search sealed packages constructed of multiple layers of packaging, different packing materials, and contents. The lack of training that replicates operationally realistic concealment in parcels limits dog teams’ capabilities and operational effectiveness.

The improvement in explosives detection from the limited intervention training suggests that deficiencies in parcel search performance may be improved through structured, progression-based training that emphasizes odor recognition under realistic operational conditions. Training plans should expose the dogs to a variety of parcel types, packaging materials, contents, and odor concentrations while progressively increasing search difficulty. By following a systematic training plan, the dogs’ ability to generalize to different odor thresholds, their search priority of productive areas, and their experience when searching parcels may be improved. However, because the study did not include a control group, the observed improvements cannot be directly attributed to the intervention.

The low detection sensitivity observed in the baseline assessment highlights the potential risks associated with inadequate training and preparation of dogs to detect explosives in parcels. In real-world operational settings, such errors would have significant safety consequences. The results of the intervention training suggest that these failures are not indicative of a dog’s inability to detect the target odor but may instead reflect limitations in odor recognition, such as lower odor thresholds or insufficient training on operationally representative search problems.

The study’s results support establishing parcel search training progression plans, odor recognition testing, and maintenance training representative of real-world operational deployments.

### 4.5. Limitations

One limitation of this study was the relatively small sample size of 13 dog teams, which limited statistical power. Consequently, the results should be interpreted as exploratory, and a larger study is needed to provide more precise estimates of detection improvement. Although the overall improvement in detection performance was statistically significant, the improvements observed for C4 and TNT individually did not reach statistical significance. The limited sample size also prevented a more detailed evaluation of potential differences associated with training location, dog experience, and other factors that may influence detection performance. Therefore, this study should be considered a preliminary proof-of-concept investigation, and larger studies involving more dog teams will be necessary to confirm these findings. Additionally, the study examined the immediate outcomes of the training intervention, but no long-term retention checks were conducted; thus, it is unclear whether the improvements observed hold over time without reinforcement, which warrants future research.

The study did not use a control group; therefore, the contribution of the training intervention cannot be independently established. The improvement in search behavior and detection sensitivity was consistent with the intended objectives of the training intervention, alternative explanations, such as repeated exposure to the target odor, familiarity with the assessment conditions, and additional encounters with the target odors, may have contributed to the success. Future research should incorporate a control group.

Another limitation was the unexpected lack of TNT recognition demonstrated by most dog teams during the ORT. Additional training was required to establish TNT recognition before the teams could proceed with the planned sealed-parcel intervention, reducing the time available for the intervention itself. Consequently, the improvements in TNT detection may reflect both increased familiarity with the target odor and improvements in parcel-search behavior. Furthermore, because the study did not include an untrained control group, the effects of the intervention cannot be fully separated from those of repeated testing or additional exposure to the target odors.

Finally, although detection sensitivity increased following training, false-positive responses also increased. This suggests that the intervention may have influenced the balance between sensitivity and specificity, potentially increasing the likelihood of responses to non-target odors. While improved sensitivity is particularly important for explosive detection applications, increased false-positive responses may have operational consequences. Future studies should evaluate whether the improvements in sensitivity can be maintained while reducing false-positive responses through additional discrimination training and extended practice.

## 5. Conclusions

The study provides preliminary evidence that less than one day of structured, odor-recognition-focused training may improve a detection dog team’s performance in carrying out sealed parcel searches. The results suggest that it is not sufficient to rely solely on the dog’s ability to recognize the odor; they should also be able to work efficiently and methodically when searching for odors emitting small amounts of vapor. A progressive and systematic training approach may be of benefit in understanding the complexities of searching sealed parcels, starting with basic odor recognition and progressing to more complex searches that replicate actual scenarios in which sealed parcels will be searched.

After combining TNT imprinting post-ORT and the sealed parcel search intervention, the dogs’ performance improved in the post-training parcel search assessment. Most dogs required the extra TNT recognition training, so the gains with TNT cannot be attributed specifically to the parcel intervention. All dogs detected C4 during the ORT, and the improved detection of C4 in sealed parcels during the post-baseline assessment is consistent with the possible effect of the parcel intervention contributing to the improvement, though repeated practice could not be ruled out.

These findings support further evaluation of the completion of an odor recognition test prior to detection training and developing a progressive training plan that covers both individual target odors and sealed parcel searches. More broadly, this study highlights the importance of evaluating canine detection performance under realistic operational conditions rather than relying exclusively on standardized certification outcomes. However, controlled studies with larger sample sizes are needed to isolate the effects of individual training components, establish the generalizability of these findings, and determine whether observed improvements translate into sustained operational performance. Future research should prioritize standardized, evidence-based training and assessment approaches that can improve the consistency, reliability, and operational readiness of detection canine teams.

Causality was not established by the baseline and post-training assessment; sealed parcel search and the observed changes could be a result of repeated testing, familiarity with the testing environment, and handler influence. The findings should be viewed as preliminary and provide support for training programs.

### 5.1. Practical Applications

These results provide several useful insights for dog training programs, specifically in parcel detection, but could also be applied to other aspects of an Explosive Detection Dog’s training.

First, training and assessment programs should include an ORT to provide a means of distinguishing between true odor recognition deficits and performance issues with regard to searching for a target during operational searches, as a dog that is unable to find a target may not necessarily have a deficit in its ability to recognize a target odor; it may in fact be due to the dog’s choice of search strategy, the threshold of sensitivity, the environmental conditions of the search, or the dog’s failure to generalize to the search scenario.

Second, training exercises should include progressively more complex and sealed-parcel, or wrapped-package, searches that become increasingly difficult. Training should reduce the amount of odor available to the dog while increasing the difficulty level of the search. The purpose of such training is to present the dog with different (1) types of packaging material, (2) package contents, and to (3) different levels of odor permeation from each parcel. This type of training will improve the dog’s methodical search behavior, increase the dog’s persistence in searching for a target, and train the dog to identify very low levels of target odor emanating from seams and/or corners of a parcel.

Ultimately, organizations should not assume they are operationally ready based on successful certifications, with no regard for conventional search environments. Demonstrated performance in one search may not be indicative of the ability to conduct searches effectively in others. Detection capability must be assessed based on the specific operational challenges posed by working dog teams, such as operating in environments with low odor thresholds from the source.

### 5.2. Future Research

While this work presents findings that a brief, focused training program may improve sealed parcel search functionality, many aspects require follow-up research.

Future research should examine how often maintenance training for the learned behaviors is needed to sustain performance improvements. It should also assess how long the performance gains from the initial training event persist (weeks or months), as well as the training frequency needed to maintain operational ability to perform searches on sealed parcels.

Future studies should also investigate how different packaging materials and contents affect the dog’s ability to detect odors. Sealed parcels can be constructed with widely different materials, internal packaging, physical dimensions, and odor permeability—all of which may influence the quantity of available odor, and thus the detection results of the dog. A good understanding of how variation in the construction and contents of the parcel affects odor availability and the results of dog detection can improve training quality and prepare the individual and/or unit for deployment.

In addition to packaging type and contents, subsequent studies should also investigate environmental influences. Often, the conditions in the search area, including ambient temperature, humidity, wind direction and speed, and search orientation, can profoundly affect how odor from sealed parcels migrates into the air, making it available to the dog. By controlling these variables in a laboratory setting for investigation, the resulting data will assist in establishing guidelines for training and conducting operational searches on sealed parcels.

Lastly, applying a double-blind validation protocol may be beneficial for future research to further reduce the risk of handler influence and build confidence in results from completed studies. The use of double-blind assessments, in addition to being a rigorous evaluation of dog performance, may demonstrate that the increase in performance was due to the training intervention (rather than inadvertent cueing or bias) and therefore provide a stronger scientific basis for operational parcel search programs.

Collectively, these areas of research may provide a stronger basis for developing more effective training methods and for providing a stronger scientific basis for operational parcel search programs.

## Figures and Tables

**Table 1:** Defined terminology used to record a dog team’s detection performance in an assessment.

Term	Recorded When…
True Positive	A parcel that did contain a target odor was correctly identified by a handler due to their dog alerting to or having a CoB to it
False Positive	A parcel that did not contain a target odor was incorrectly identified by a handler due to their dog alerting to or having a CoB to it
True Negative	A parcel that did not contain a target odor was correctly identified as “blank” by a handler due to their dog not alerting to or having a CoB to it
False Negative	A parcel that did contain a target odor was incorrectly identified as “blank” by a handler due to their dog not alerting to or having a CoB to it

**Table 2:** Parcel Assessment Performance (*n* = 7 for Lubbock; *n* = 6 for Miami).

Location	Explosive	Assessment	Sensitivity (%)	Specificity (%)
Lubbock	C4	Baseline	57	91
		Post-Training	71	84
	TNT	Baseline	29	91
		Post-Training	57	84
Miami	C4	Baseline	17	100
		Post-Training	67	90
	TNT	Baseline	0	100
		Post-Training	50	90

Sensitivity = TP/(TP + FN) × 100; specificity = TN/(TN + FP) × 100, where TP = true positive, FN = false negative, TN = true negative, and FP = false positive.

**Table 3:** Paired comparisons of baseline and post-training explosive detection performance (*n* = 13 dog teams).

Comparison	Baseline Sensitivity	Post-Training Sensitivity	Change (pp)	Statistical Test	Exact *p*-Value
Overall Detection	27% (7/26)	61% (16/26)	+34.6	Exact paired sign test	0.0078
C4 Detection	38% (5/13)	69% (9/13)	+30.8	Exact McNemar’s test	0.125
TNT Detection	15% (2/13)	54% (7/13)	+38.5	Exact McNemar’s test	0.0625

Sensitivity = TP/(TP + FN) × 100. Change is the absolute percentage-point difference. All *p*-values are two-sided. The overall sign test compares the number of targets detected per team (0–2); eight teams improved, five were unchanged, and none worsened. Exact McNemar’s tests compare paired detection outcomes for each explosive.

## Data Availability

The raw data supporting the conclusions of this article will be made available by the authors on request.

## Appendix A

**Table A1:** Details of all alerts from each dog team, across both locations, during the ORT, Baseline Parcel, and Post-Training Parcel searches.

		Lubbock Teams							Miami Teams					
		1	2	3	4	5	6	7	1	2	3	4	5	6
**ORT**	C4 True Positives	1	1	1	1	1	1	1	1	1	1	1	1	1
	TNT True Positives	1	0	0	0	0	0	0	0	0	0	0	1	0
	False Positives	0	1	0	0	0	1	0	1	1	3	0	2	1
**Baseline Parcels**	C4 True Positives	0	1	0	1	0	1	1	0	1	0	0	0	0
	TNT True Positives	0	0	1	1	0	0	0	0	0	0	0	0	0
	False Positives	0	0	0	1	0	0	4	0	0	0	0	0	0
**Post-Training Parcels**	C4 True Positives	1	1	0	1	0	1	1	0	1	0	1	1	1
	TNT True Positives	0	1	1	1	0	1	0	1	0	1	1	0	0
	False Positives	3	1	2	0	2	0	1	0	1	2	0	1	1
